# Structure of the human TRiC/CCT Subunit 5 associated with hereditary sensory neuropathy

**DOI:** 10.1038/s41598-017-03825-3

**Published:** 2017-06-16

**Authors:** Jose H. Pereira, Ryan P. McAndrew, Oksana A. Sergeeva, Corie Y. Ralston, Jonathan A. King, Paul D. Adams

**Affiliations:** 1 Molecular Biophysics and Integrated Bioimaging Division, Lawrence Berkeley National Laboratory, Berkeley, CA 94720 USA; 2 Biology Department, Massachusetts Institute of Technology, Cambridge, MA 02139 USA; 3 Berkeley Center for Structural Biology, Lawrence Berkeley National Laboratory, Berkeley, CA 94720 USA; 4 Department of Bioengineering, University of California Berkeley, Berkeley, CA 94720 USA

## Abstract

The human chaperonin TRiC consists of eight non-identical subunits, and its protein-folding activity is critical for cellular health. Misfolded proteins are associated with many human diseases, such as amyloid diseases, cancer, and neuropathies, making TRiC a potential therapeutic target. A detailed structural understanding of its ATP-dependent folding mechanism and substrate recognition is therefore of great importance. Of particular health-related interest is the mutation Histidine 147 to Arginine (H147R) in human TRiC subunit 5 (CCT5), which has been associated with hereditary sensory neuropathy. In this paper, we describe the crystal structures of CCT5 and the CCT5-H147R mutant, which provide important structural information for this vital protein-folding machine in humans. This first X-ray crystallographic study of a single human CCT subunit in the context of a hexadecameric complex can be expanded in the future to the other 7 subunits that form the TRiC complex.

## Introduction

The eukaryotic chaperonin TRiC (T-complex protein-1 Ring Complex, also known as CCT: Chaperonin Containing T-complex protein-1) plays an important role in ensuring efficient folding of nascent or stress-denatured proteins^1^. TRiC interacts with approximately 10% of the entire proteome and its function is absolutely essential for viability of the cell^2^. The cellular accumulation of misfolded protein has been associated with several human diseases, including Alzheimer’s disease, Huntington’s disease, and cancer, making TRiC a potential therapeutic target^3, 4^.

The overall structural arrangement of chaperonins is of two rings of seven to nine subunits each stacked back-to-back, creating a large cylindrical protein complex of approximately 1 MDa. Chaperonins can be classified into two major groups. Group I chaperonins, such as GroEL from *Escherichia coli*, are found in prokaryotes and eukaryotic organelles, and generally consist of double homo-heptameric ring arrangements^5^. In contrast, group II chaperonins exist in the archaeal and eukaryotic cytosol and consist of double hetero (or homo)-octameric or nonameric rings^6^. Group I chaperonins use a homo-heptameric ring-shaped cofactor (GroES in the case of GroEL) to fully close the protein folding chamber, whereas group II chaperonins contain a “built-in” lid formed by an extended α-helix at the tip of the apical domains^7^.

Human TRiC belongs to the group II chaperonin family and consists of two hetero-octameric rings containing eight distinct subunits (CCT1-8)^8, 9^. For more than 2 decades, group II chaperonins have been studied structurally in order to better understand the ATP-dependent folding mechanism of this class of proteins. The majority of the crystal structures available are of the simpler TRiC-like complexes consisting of rings that have one to three different subunits from archaeal organisms^6, 10–15^. More recently, low-resolution structures of the hetero-oligomeric complex of TRiC from bovine^16, 17^ and yeast systems^17–20^ have been solved showing large asymmetric features when comparing the subunits. However, to date no crystallographic data have been available for the full human TRiC complex or even a single subunit. In this paper, we describe the crystal structure of the human CCT5 subunit. The sequence similarity between CCT5 and others TRiC subunits (from 32 to 40% identity) allows us to understand important structural regions such as the nucleotide-binding site and substrate-recognition sites of the different subunits. In addition, the CCT5 subunit is of special interest because the mutation of histidine-147 to arginine (H147R) in the human CCT5 gene is associated with autosomal recessive mutilating sensory neuropathy with a spastic paraplegia disease^21^. The crystal structure of human CCT5 reveals a classical chaperonin complex arrangement with two back-to-back rings of eight subunits each, similar to the organization of the full TRiC complex^19^. Moreover, the human CCT5 homo-oligomeric complex is able to hydrolyze ATP and refold misfolded protein substrates^22^. Interestingly, this CCT5 homo-oligomeric complex is also able to suppress the mutant huntingtin (mHTT) protein aggregation associated with Huntington neurodegenerative disease^23^. Therefore, the crystal structure of human CCT5 subunit provides information not only about the structural features of TRiC but also advances our knowledge that will be valuable for therapeutic design.

## Results and Discussion

### Human CCT5 Subunit Architecture

**Figure 1:**
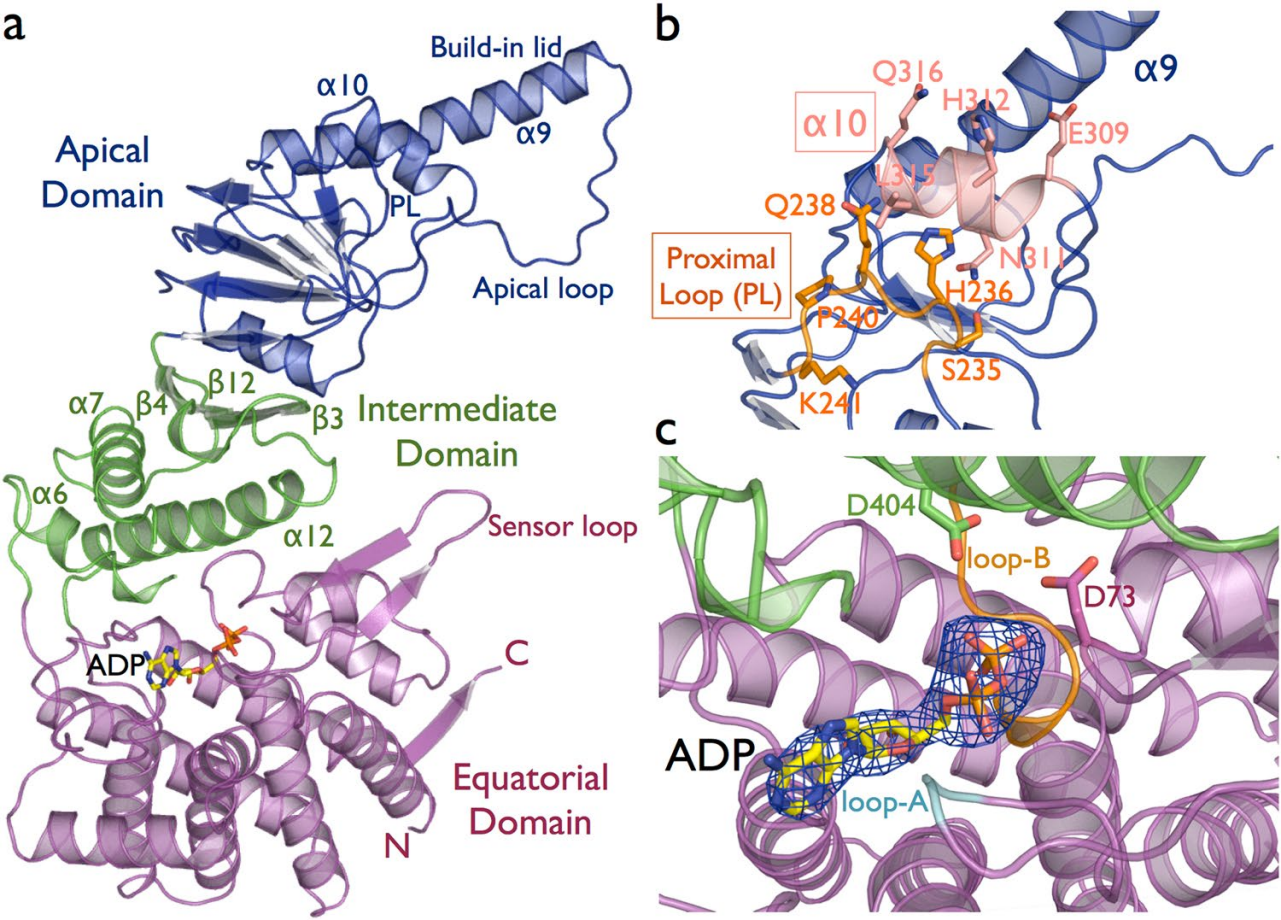
(**a**) Cartoon representation of human CCT5 subunit architecture in complex with ADP nucleotide. The N-terminal and C-terminal regions of the subunit form the equatorial domain, which includes the nucleotide-binding site and sensor loop. The intermediate domain that connects the equatorial and apical domains via the lower hinge is composed of α6, α7 and α12, and the upper hinge represented by the β-sheet β3/β4/β12. The apical domain is associated with the substrate binding recognition and with the substrate-folding process though the apical loop region. In addition, the extended α9 serves as a lid to close the TRiC ring chamber. (**b**) The substrate-binding interface has been identified between the α10 helix in and the proximal loop (PL) region^28^. In CCT5, the α10 is formed by the residues _309_EANHLLLQ_316_ and the proximal loop by the residues _234_FSHPQMPK_241_, with surface-exposed residues represented in sticks. (**c**) A mF_o_-DF_c_ electron density map omitting the ADP nucleotide molecule contoured at 3.0 σ is shown in blue. The nucleotide-binding site shows the loop-A and the loop-B surrounding the phosphate groups of ADP. The ATP hydrolysis occurs by a water nucleophilic attack, and the water molecule is held in place by the represented residues D73 and D404.

The general subunit architecture of the CCTs subunits comprise three distinct domains: an equatorial domain that includes the nucleotide-binding site, an apical domain involved in substrate binding, and an intermediate domain that connects the equatorial and apical domains via two hinge regions (Fig. 1a). The CCT5 equatorial domain is formed of the N-terminal (residues 1–154) and the C-terminal (residues 418–541) regions. This domain contains two nucleotide-binding loops that interact with phosphate groups and the adenine ring of ADP. The loop-A residues Gly-53 and Pro-54 interact with the α-phosphate group and adenine ring of ADP, respectively. Loop-B - _103_GDGTTG_108_ - contains the classical P-loop motif, where threonine residues coordinate the β-phosphate group of ADP (Fig. 1c). The proposed mechanism of ATP hydrolysis occurs through water nucleophilic attack, in which the water molecule is held in place by residues Asp-73 and Asp-404 in CCT5 based on structural studies of the Thermosome^12^. The intermediate domain contains two hinges formed by residues 155–226 and 381–417. The lower hinge contains exclusively α-helices, in which helix-6 and helix-12 make contact with the equatorial domain, and helix-7 contacts the upper hinge, which is composed of the β-sheet β3/β4/β12. Experiments in the group II chaperonin Mm-Cpn from *Methanococcus maripaludis* ^24^ and the group I chaperonin GroEL^25, 26^ show that ATPase activity is abolished when mutations are made to the residue equivalent to Asp-404 in CCT5, located in helix-12 of intermediate domain. The apical domain is composed of residues 227 to 378 and shows the most sequence divergence between the human TRiC subunits and is associated with the substrate recognition^27, 28^. The hetero-octameric ring formed by eight specialized subunits may lead to a large diversification of proteome targets in eukaryote evolution^2, 29^. The substrate-binding interface has been identified in mouse and yeast CCT as being between helix-11 (equivalent to helix-10 in CCT5) and the proximal loop (PL) region^28^ (Fig. 1b). In CCT5, helix-10 consists of the residues _309_EANHLLLQ_316_ and the proximal loop of the residues _234_FSHPQMPK_241_ (Supplementary Fig. 1). Above the region of the substrate-binding site, the apical domain possesses an extended α-helix (helix-9), which serves as a built-in lid to close the TRiC folding cavity.

**Figure 2:**
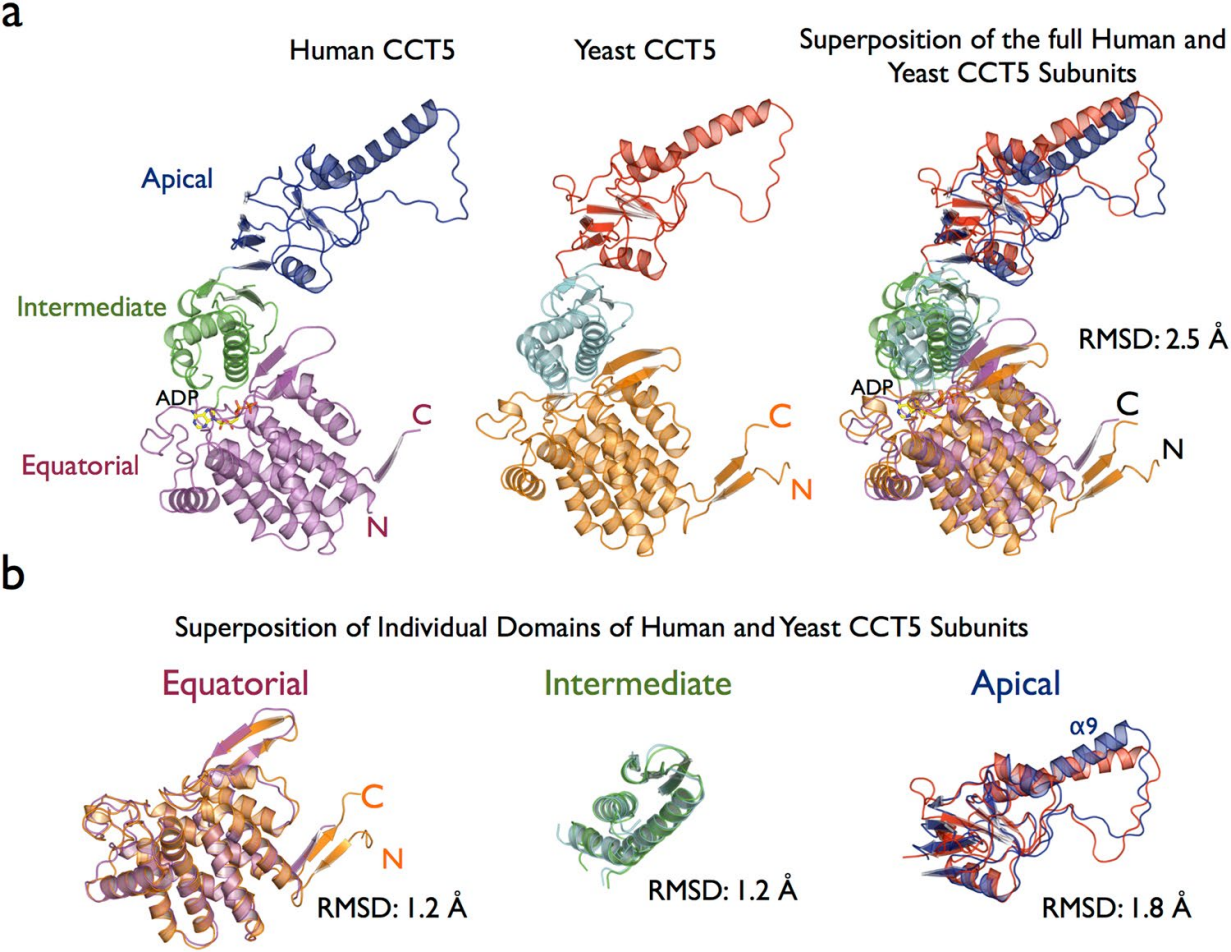
(**a**) Cartoon representation of the single subunits of human CCT5 and yeast CCT5 (PDB ID 4V8R)^18^ and a superposition between human CCT5 and yeast CCT5. (**b**) Individual superposition of the equatorial, intermediate and apical domains from human CCT5 and yeast CCT5 showing that the structural similarity between them. The highest RMSD for the individual domains was observed for the apical domain because of the local conformation change in the built-in lid region (α9) involved in closing the TRiC folding cavity.

Large protein complexes composed of several subunits, which are organized into distinct domains, are not expected to necessarily form well diffracting crystals because of their intrinsic flexibility. The group II chaperonin protein family is no exception, with the highest resolution of crystallographic data collected to date for a eukaryotic group II chaperonin being 3.8 Å for yeast TRiC^17–20^ and 5.5 Å resolution for bovine TRiC^16, 17^. The human CCT5 and CCT5-H147R subunits were solved at 3.5 Å and 3.6 Å resolution, respectively (Table 1). In order to compare the human CCT5 single subunit with other eukaryotic chaperonins, we selected the highest resolution structure with the lowest R_free_ value deposited in the Protein Data Bank. The human CCT5 subunit was superposed with chain E of the yeast TRiC structure, which corresponds to the CCT5 subunit in yeast (PDB ID 4V8R)^18^. The sequence identity between the human CCT5 and yeast CCT5 subunits is 60%, which leads to a similar overall subunit structural architecture. Superposition of the human CCT5 and yeast CCT5 structures using Cα coordinates has a RMSD in coordinate position of 2.5 Å. This is a result of the rotation of the individual domains rather than large local conformation differences within the individual domains (Fig. 2a). Rotations of the intermediate and apical domains are associated with protein folding cycle related to nucleotide states^10, 11, 30^. Individual superposition of the Cα coordinates of the equatorial, intermediate and apical domains from human CCT5 with yeast CCT5 have RMSDs of 1.2 Å, 1.2 Å and 1.8 Å, respectively, confirming the structural similarity between the individual domains of the eukaryotic group II chaperonins. The highest RMSD observed for apical domain between human CCT5 and yeast CCT5 is related the local conformation change in the built-in lid (α-helix9) used to close the TRiC folding cavity (Fig. 2b).

**Table 1:** Statistics for data collection and refinement of CCT5 and CCT5-H147R structures.

	CCT5	CCT5-H147R
***Data collection***		
Wavelength (Å)	1.000	1.000
Resolution range (Å)	75.7–3.5 (3.59–3.5)	65.0–3.6 (3.66–3.6)
Detector Distance (mm)	400	450
Φ (deg.) collected/ΔΦ (deg.)	180/0.5	200/0.2
Exposure time (seconds)	3	3
Temperature of collect (Kelvin)	100	100
***Data statistics***		
Space group	P 4 21 2	P 4 21 2
Unit-Cell parameters (Å)	a = 204.71, b = 204.71 and c = 162.95	a = 205.57, b = 205.57 and c = 163.55
Total reflections	584723 (54296)	606725 (60364)
Unique reflections	44068 (4305)	41130 (4031)
Multiplicity	13.3 (12.6)	14.7 (14.6)
Data completeness (%)	100 (99.0)	100 (100)
I/σ(I)	13.7 (1.2)	10.9 (1.3)
R_*sym*_^a^	0.223 (2.32)	0.297 (2.41)
CC1/2	0.998 (0.355)	0.997 (0.357)
***Structure Refinement***		
R_*factor*_^b^ (%)	26.1	27.5
R_*free*_^c^ (%)	31.7	32.7
RMS from ideal geometry		
Bond lengths (Å)	0.003	0.003
Bond angles (º)	0.885	0.779
Protein residues per AU^d^	2064 (4 subunits)	1934 (4 subunits)
ADP	4	4
Ramachandran Plot		
Favored region (%)	83.7	82.7
Outliers region (%)	2.9	3.7
Clashscore	6.5	7.67
Molprobity Score	2.3	2.6
Percentile	99	97
Rotamer outliers	2.1	4.4

^a^R_*sym*_ = Σ_*hkl*_ Σ_*i*_|*I*_*i*_ (*hkl*) − 〈*I* (*hkl*)〉 |/Σ_*hkl*_ Σ_*i*_ *I*_*i*_ (*hkl*), where Σ_*hkl*_ denotes the sum over all reflections and Σ_*i*_ is the sum over all equivalent and symmetry-related reflections.

^b^R_*factor*_ = Σ |Fobs − Fcalc|/Σ Fobs.

^c^R_*free*_ = R_*factor*_ for 5% of the data were not included during crystallographic refinement.

^d^AU = Asymmetric unit.

### Human CCT5 Subunit shows a TRiC-like arrangement

**Figure 3:**
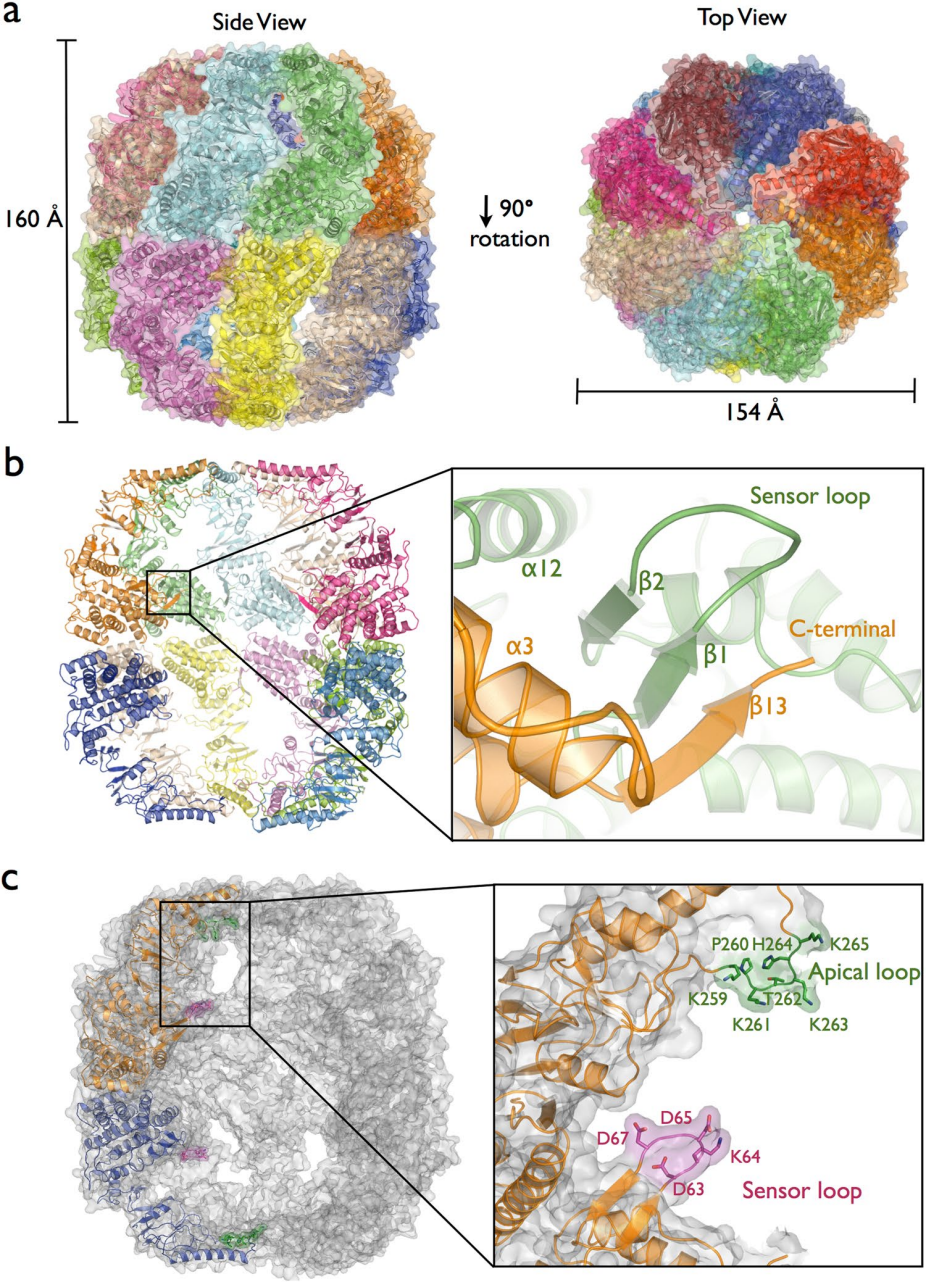
(**a**) Side and top view using a cartoon and surface representation for each of the human CCT5 subunits forming the double-rings homo-octameric shape similar to TRiC-like arrangement. The CCT5 complex was observed in the closed state of group II chaperonin characterized by intra-ring contacts between all the three domains; equatorial, intermediate and apical. Apical domains serve as a lid of the rings creating a protected environment for protein folding. (**b**) Intra-rings contacts at the equatorial domain form a β-sheet element by β1 and β2 of one subunit and β13 from the adjacent subunit. This β-sheet element is conserved in all group II chaperonin structures in both closed and open states indicating these intra-ring interactions are important for maintain the ring assemble. (**c**) Position of the sensor loop and the apical loop in the context of the CCT5 homo-octameric rings. The sensor loop residues _63_DKDGD_67_ and the apical loop residues _259_KPKTKHK_265_ are point into to cavities creating highly charged regions. These loops have been associated with substrate interaction from bovine TRiC structure^16^.

The CCT5 homo-oligomeric complex has the two back-to-back rings of eight subunits similar to the full human TRiC complex (Fig. 3a and Supplementary Fig. 2a–e). A previous cryo-electron microscopy (cryo-EM) study had shown that subunits CCT4 and CCT5 each retain the ability to form active homo-octameric double ring oligomers^22, 23, 31^. Evolutionary tree analysis of the CCT subunits has shown that the human CCT4 and CCT5 subunits are most closely related to the more primitive TRiC-like chaperonins from the archaea *Thermoplasma acidophilum* and *Sulfolobus solfataricus* ^32, 33^. This sequence similarity could be the reason why out of the eight subunits only CCT4 and CCT5 retain the ability to form rings from a single subunit. However, there is no evidence that these CCT4 and CCT5 homo-oligomeric complexes are present in human cells. Despite the fact of that some studies have suggested that human TRiC could have a variable subunit composition and this may be related to chaperonin function^34, 35^, a precisely ordered arrangement within the rings has become more generally accepted for all eight TRiC subunits^17, 20, 36^.

The arrangement of the subunits in the TRiC complex has been a source of much debate and study^19, 37–39^. The latest TRiC subunit arrangement from two independent cross-linking studies, using yeast and bovine chaperonins, proposed that the subunits order anti-clockwise 1-3-6-8-7-5-2-4 for the upper ring, contacting the lower ring subunits 7–8-6-3-1-4-2-5, respectively^17, 20^. This proposed arrangement places the equatorial domain of the CCT5 subunit in direct contact with the equatorial domain of the CCT4 subunit of the opposite ring. This, combined with the observation that both CCT4 and CCT5 subunits are able to spontaneously form double homo-octameric ring complexes when recombinantly expressed in *E. coli* ^22, 23, 31^, suggests that they share similar ring-ring interfaces, which form stable ring interface interactions. It is possible that the structure of the CCT5 homo-oligomeric complex is representative of an ancestral TRiC chaperonin. Over time, the subunits have become specialized, with accumulated mutations in the substrate binding regions defining the potential substrates, and mutations in the subunit-subunit interfaces defining the key interactions that dictate the ordering of subunits around the ring. Thus the sequence diversity between the subunits of the hetero-octameric TRiC is not only important for the increase in the range of substrates in eukaryotes^2^, but also critical to defining the specific inter-ring and intra-ring subunit interactions that form a stable complex.

### Human CCT5 in a complex with ADP nucleotide in a closed state conformation

The binding and hydrolysis of ATP has been shown to promote the transition from a substrate-binding acceptor state (open state) to a folding-capable state (closed state)^24^. We have solved the CCT5 homo-oligomeric complex in the closed conformation, which shows intra-ring contacts between the equatorial, intermediate and apical domains (Fig. 3a). In contrast, in the open conformation of the Mm-Cpn chaperonin, only the equatorial domain shows intra-ring contacts (Supplementary Fig. 3). The equatorial domain is the largest domain and is involved in contacts between the upper and lower rings and contacts between adjacent subunits in the same ring. A region of particular interest in the equatorial domain is the sensor loop, which makes contact with the substrate tubulin in the bovine CCT structure^16^. The sensor loop (also called stem-loop)^40, 41^ is located between β1 and β2 in CCT5 and it is composed of residues _63_DKDGD_67_, where the DXXGD motif is observed for all 8 human CCT subunits (Supplementary Fig. 1). The sensor loop residues side-chains point into the folding cavity and present a negatively charged surface for interaction with the potential encapsulated unfolded or partially folded substrate (Fig. 3c). In the homo-octameric CCT5 ring, the stem loop β-strands, β1 and β2, interact with the C-terminal β-strand, β13, of an adjacent subunit (Fig. 3b). A similar β-sheet formation between adjacent subunits has been previously observed in archaeal chaperonin structures^10, 11, 42^. The formation of this β-sheet between adjacent subunits suggests that these contacts are essential for the intra-ring interface observed for the group II chaperonins. However, the adjacent subunit of the archaeal chaperonin involves both N-terminal and C-terminal β-strands, whereas for CCT5 only the C-terminal β-strand, β13, was observed to be involved.

In addition to the sensor loop region located at the equatorial domain, a region in the apical domain has been implicated in substrate binding inside the TRiC cavity. The tubulin substrate is seen to make contact with an apical loop region^16^. In CCT5 this loop consists of the residues _259_KPKTKHK_265_, which is highly basic because of the large number of lysine residues (Fig. 3c). The charged and hydrophilic nature of the interior of the chaperonin cavity, such as observed for the CCT5 sensor loop and apical loop, have been linked to the mechanism of folding substrate in GroEL^43^.

As mentioned previously, the substrate-binding interface has been identified as lying between helix-10 in CCT5 and the proximal loop region^28^. ATP hydrolysis closes the ring bringing this substrate-binding interface in the apical domain into contact with a loop region of the adjacent subunit. This intra-ring contact between the apical domains of adjacent subunits has been proposed to displace the substrate from its binding-site and release it inside the cavity to be folded. Thus the loop from the adjacent subunit was called “release loop of substrate or rls loop”^11, 30^. Analyzing the CCT5 sequence and the ring arrangement, the rls loop corresponds to residues _343_PRFSELT_349_ and indeed makes contact with the substrate-binding interface from the adjacent subunit (Supplementary Fig. 4).

### CCT5-H147R mutant structure

A mutation in human CCT5 subunit at position 147 that replaces a histidine with an arginine residue has been implicated in hereditary sensory neuropathy, which is a class of disorder marked by degeneration of the nerve fibers in the sensory periphery neurons^21^. However, how this mutation leads to the disease phenotype is still unknown^44^. Sergeeva and collaborators showed the CCT5-H147R mutant has reduced folding activity compared to CCT5-WT against human γD-crystallin and actin substrates. It has been hypothesized that decreased protein-folding activity by TRiC complexes containing the CCT5-H147R mutant subunit compared to TRiC-WT could have an impact on many protein substrates, such as tubulin and actin, which seems very plausible^31^. Since neurons have a large abundance of microtubules, tubulin is a good candidate for a substrate that is adversely affected by the CCT5-H147R mutation and therefore leads to the disease phenotype^45^.

**Figure 4:**
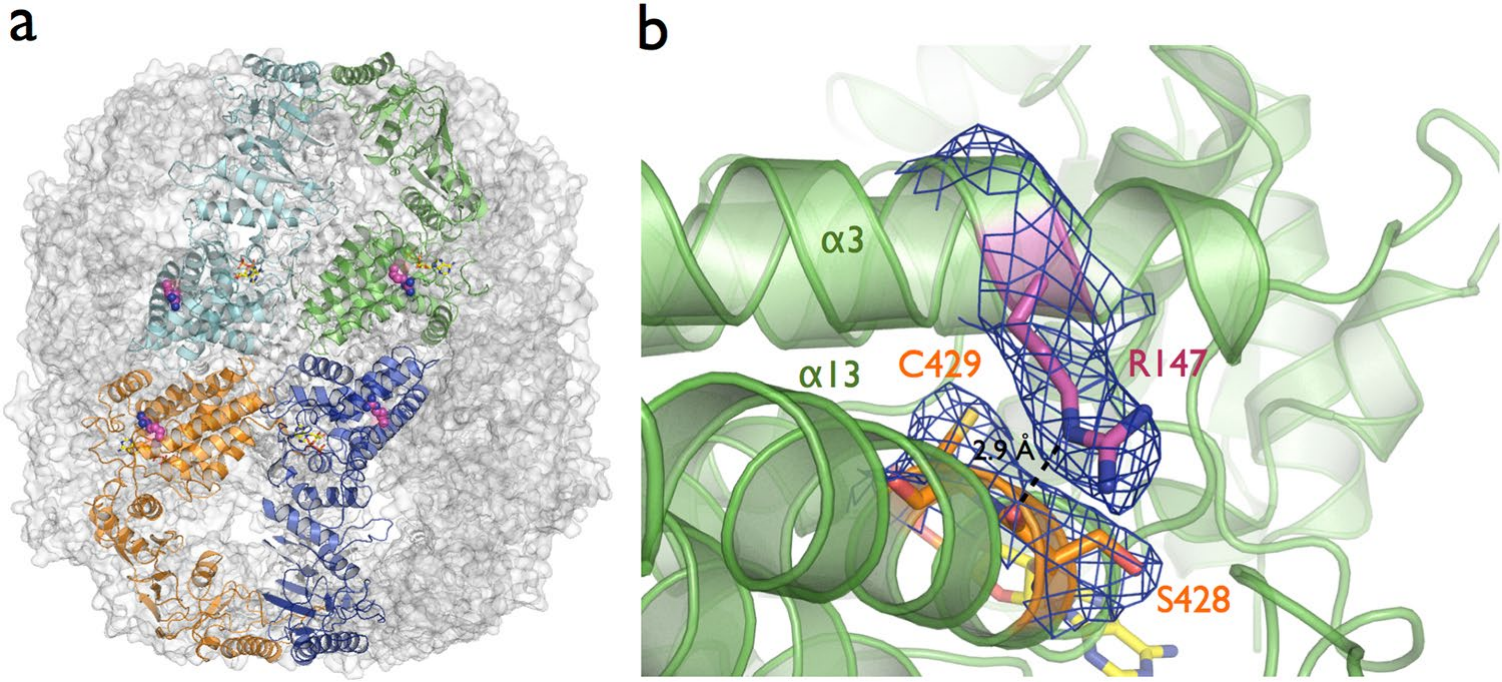
(**a**) Mutant CCT5-H147R shows the same double homo-octameric ring arrangement as CCT5-WT. The CCT5-H147R structure demonstrated the Arg147 residue does not point into the inter-rings or intra-ring (between subunits) interfaces. (**b**) A 2mF_o_-DF_c_ electron density map for CCT5-H147R around the side chain of Arg147 and Ser428. The broken line represent the hydrogen bond distance between NE atom of Arg147 located at helix-5 and the main carbonyl group of Ser428 located at helix-13 of equatorial domain.

A structural study using an archaeal chaperonin as a model to understand the His147Arg mutant proposed that the mutation impaired oligomeric assembly and ATPase activity^46^. However, biochemical analysis of CCT5-H147R showed that it was still able to hydrolyze ATP and also refold protein substrates^31^. Our crystal structure of the CCT5-H147R mutant shows the same double homo-octameric ring arrangement as CCT5-WT indicating that the mutation does not affect the oligomeric assembly capability of the homo-octameric ring of the CCT5-H147R subunit (Fig. 4a). The structure also shows that the Arg147 residue is not positioned in a way that would disrupt the inter-ring or intra-ring interfaces, therefore it is unlikely that the mutation would impair the oligomeric assembly of the full TRiC complex containing the CCT5-H147R subunit (Fig. 4a). In addition, the CCT5-H147R structure was solved in complex with the nucleotide ADP showing the mutant still can bind nucleotide like the wild-type CCT5. This is consistent with the observation that the CCT5-H147R and CCT5-WT subunits have similar ATP hydrolysis rates^31^.

The electron density map for CCT5-H147R demonstrates clear density for the side chain of Arg147, and shows that it is positioned to make a hydrogen bond between the NE atom of Arg147 located on helix-5 and the main carbonyl group of Ser428 located at helix-13 of the equatorial domain (Fig. 4b). Significant conformation changes, such as rotation and local movement of the equatorial domain are associated with ATP hydrolysis and the folding mechanism^10, 11, 16^. Changes in contacts between structural elements in the complex could have an impact on the flexibility within the equatorial domain, thus modulating the function of a particular subunit. Since TRiC is a highly allosteric molecular machine^24, 47–49^, the CCT5-H147R point mutation could have an impact on the function of the entire complex by changing cooperativity within the complex.

## Conclusion

The CCT5 subunit structure reveals, in the highest resolution to date for a eukaryotic chaperonin, important regions in the human TRiC complex, such as the nucleotide-binding site, substrate-binding site, sensor loop, apical loop and the release-loop of the substrate. The structures of the single CCT5 and CCT5-H147R subunits show the ability to form the double ring octameric arrangement similar to the human TRiC. In agreement with previous biochemical studies using the CCT5 homo-oligomeric complex^31^, both structures of the CCT5 and CCT5-H147R homo-oligomeric in complex with nucleotide show that the catalytic residues Asp73 and Asp404 are positioned to participate in ATP hydrolysis.

The therapeutic properties of these homo-oligomeric recombinantly expressed human CCT4 and CCT5 subunits can be explored more broadly. Previous studies have demonstrated that CCT1, CCT3 and CCT4 show specific interactions with the Q/N-rich huntingtin protein that is implicated in Huntington’s disease^50, 51^. Similarly, the CCT5 homo-oligomeric complex was also able to suppress mutant Huntingtin protein aggregation demonstrating a different phenotype when compared to the monomeric CCT5 subunit^23, 31^. The knowledge derived from the CCT5 structure described here can be extended based on sequence homology to the other subunits. Bioengineering experiments based on the CCT5 structure, such as shuffling substrate-binding sites, sensor loops and apical loops, are under investigation in order to understand the unique properties of each TRiC subunit. Switching stretches of amino acids or whole domains between the eight human CCT subunits in order to create a specific chaperone function for a health related target while keeping the equatorial and intermediate domains from CCT5 as a scaffold may generate a suite of synthetic protein-folding machines optimized for therapeutic purposes.

## Methods

### Cloning, expression, and purification of human CCT5

Cloning, expression, and purification of human CCT5 were as described previously^22^. The CCT5 plasmid was used to create the CCT5-H147R mutant. The CCT5-H147R mutant was generated using Biozilla Inc. mutagenesis service (South Francisco, CA, USA). Briefly, the pET21a-CCT5 and pET21a-CCT5-H147R constructs were transferred into BL21(DE3)RIL *Escherichia coli* expression cells. A single bacterial colony, grown on Luria-Bertani (LB) agar containing 100 µg/mL ampicillin was used to inoculate 5 mL of LB liquid culture supplemented with the same antibiotic concentrations and grown overnight at 37 °C. The overnight culture was used to inoculate a 1 L Terrific Broth culture grown at 37 °C until it reached an OD_600_ of 1.0. Expression was induced by the addition of 0.5 mM IPTG, and the culture was transferred at 20 °C and grown for 24 hours. Cell paste was lysed in 25 mM Hepes pH 7.5, 100 mM NaCl, 5 mM MgCl_2_ and 1 mM DTT and loaded onto a 5 mL Histrap column on an AKTA HPLC. The column was washed to baseline prior to elution with a gradient of 0–100% buffer 25 mM Hepes pH 7.5, 100 mM NaCl, 5 mM MgCl_2_, 1 mM DTT and 500 mM imidazole in 20 column volumes. The cleanest elution fractions were pooled and dialyzed back into the lysis buffer and applied onto a 5 mL HiTrap Q column. The cleanest elution fractions were concentrated and applied to a Size Exclusion Sephacryl S-200 column. The purified human CCT5 was concentrated to 12 mg/ml for crystallographic studies.

### Crystallization, X-ray data collection, and Structure determination

The human CCT5 and CCT5-H147R were crystallized in complex with 5.0 mM Adenosine 5′-diphosphate (ADP). The human CCT5 was screened using the sparse matrix method^52^ and the following crystallization screens: Crystal Screen, SaltRx, PEG/Ion, Index and PEGRx (Hampton Research, Aliso Viejo, CA) and Berkeley Screen (Lawrence Berkeley National Laboratory, Berkeley, CA). Crystals of CCT5 were found in 0.1 M Sodium acetate, 0.04 M Citric acid, 0.06 M Bis-Tris propane pH 6.4 and 25% (w/v) Polyethylene glycol 400. The crystals of the mutant CCT5-H147R were grown using similar crystallization conditions as the CCT5 crystals. Crystals of CCT5 and CCT5-H147R were obtained after 2 days by the sitting-drop vapor-diffusion method with the drops consisting of a mixture of 0.2 μL of protein solution and 0.2 μL of reservoir solution. Crystals of CCT5 and CCT5-H147R were soaked in a reservoir solution containing 5% (v/v) of glycerol as the cryo-protector solution. The X-ray data sets for the CCT5 and the CCT5-H147R were collected at the Berkeley Center for Structural Biology beamline 5.0.2 of the Advanced Light Source at Lawrence Berkeley National Laboratory (LBNL). The diffraction data were recorded using a Pilatus 6M detector. The data sets were processed using the program Xia2^53^. The high-resolution cutoff was set according to the methods described by Karplus and Diederichs^54^.

The crystal structure of CCT5 was solved by the molecular-replacement method using as a search model subunit 5 from the structure of yeast TRiC (PDB code 4V81)^18^ with the program Phaser^55^ within the Phenix suite^56^. The crystal structure of CCT5-H147R was solved by the molecular-replacement method using the CCT5 structure as the search model. Structure refinement was carried out using the phenix. refine program^57^. Translation-libration-screw (TLS) refinement was used, with each domain (equatorial, intermediate and apical) of each subunit identified as a TLS group. Manual rebuilding using COOT^58^ and addition of ADP nucleotide molecules allowed construction of the final models. The nucleotide-binding site was interpreted in the CCT5 and CCT5-H147R structures using cross-validated sigmaA weighted electron density maps (mFo-DFc Fourier synthesis) contoured at 3.0 σ. Root-mean-square deviation differences from ideal geometries for bond lengths, angles and dihedrals were calculated with Phenix^56^. The overall stereochemical quality of the final model for CCT5-ADP was assessed using the program MolProbity^59^. The overall statistics for X-ray data collection and structures refinement are summarized in Table 1. The atomic coordinates and structure factors of human CCT5 and CCT5-H147R mutant have been deposited in the Protein Data Bank with accession codes 5UYX and 5UYZ, respectively.

## Electronic supplementary material

Structure of the human TRiC/CCT Subunit 5 associated with hereditary sensory neuropathy
