# Supplementary material for: Structure of the human TRiC/CCT Subunit 5 associated with hereditary sensory neuropathy
Source: Sci Rep. 2017 Jun 16;7:3673. doi: 10.1038/s41598-017-03825-3 (PMC5473854; doi:10.1038/s41598-017-03825-3)
Supplement: Supplementary file 1 — Structure of the human TRiC/CCT Subunit 5 associated with hereditary sensory neuropathy [file 41598_2017_3825_MOESM1_ESM.pdf]

Supplementary Information

Structure of the human TRiC/CCT Subunit 5 associated with hereditary sensory neuropathy

Jose H. Pereira[1,2], Ryan P. McAndrew[1,2], Oksana A. Sergeeva[3], Corie Y. Ralston[1,4], Jonathan A. King[3], Paul D. Adams[1,2,5,*]

[1]Molecular Biophysics and Integrated Bioimaging Division, Lawrence Berkeley National Laboratory, Berkeley, CA 94720, USA.
[2]Joint BioEnergy Institute, Emeryville, CA 94608, USA.
[3]Biology Department, Massachusetts Institute of Technology, Cambridge, MA 02139, USA.
[4]Berkeley Center for Structural Biology, Lawrence Berkeley National Laboratory, Berkeley, CA 94720, USA
[5]Department of Bioengineering, University of California Berkeley, CA 94720, USA.

Index

	Page
**Supplementary Fig 1**	2
**Supplementary Fig 2**	4
**Supplementary Fig 3**	7
**Supplementary Fig 4**	8
**References**	8

Supplementary Fig 1

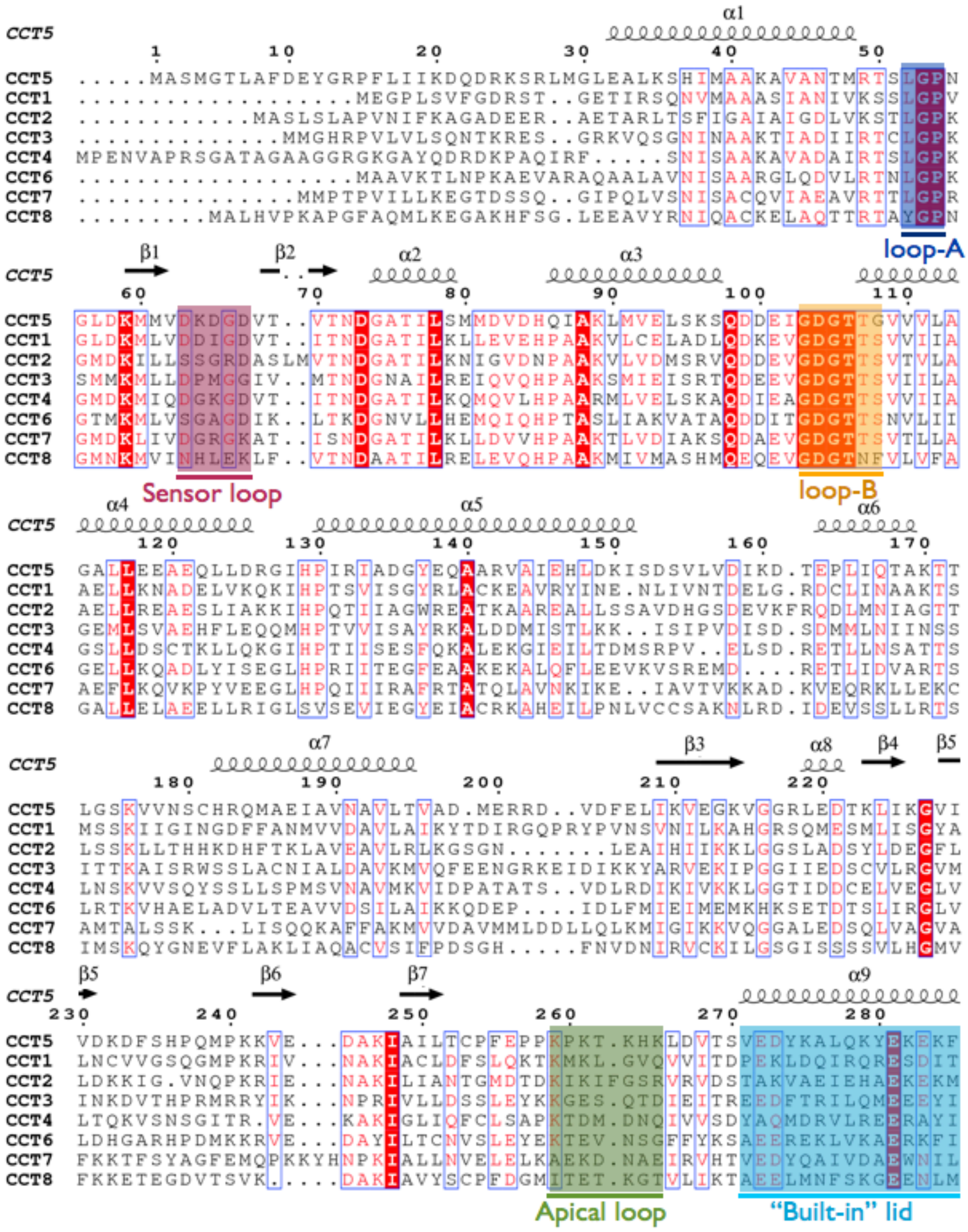

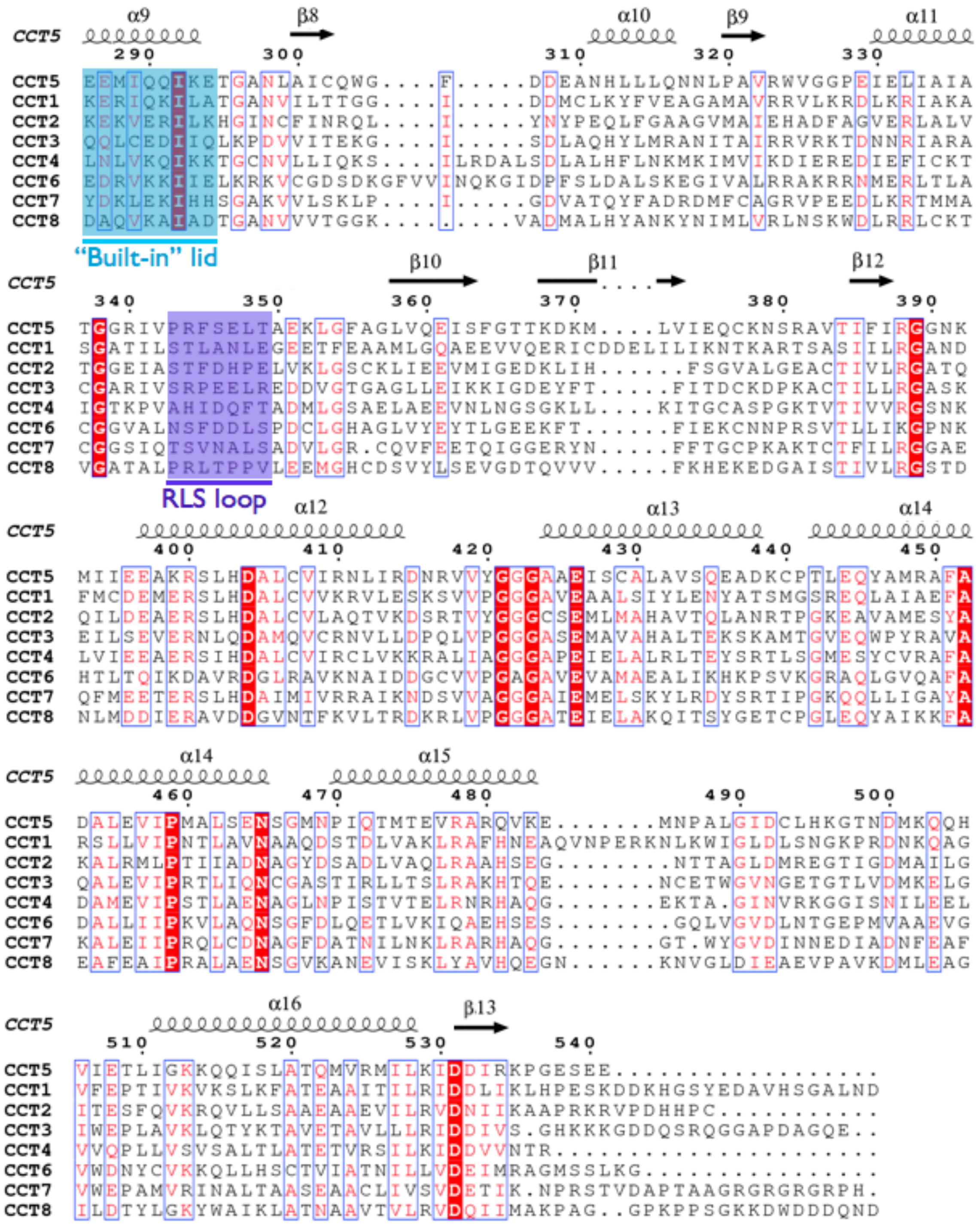

Supplementary Fig 1 – Sequence alignment of the eight human TRiC/CCT subunits. The secondary structure elements for CCT5 subunit are displayed at the top of the sequence alignment. The nucleotide-binding loop-A and loop-B, the sensor loop, rls-loop and the Apical-loop regions are indicated bellow the sequences. Figure was made using the program ESPRIPT (Robert & Gouet, 2014).

Supplementary Fig 2

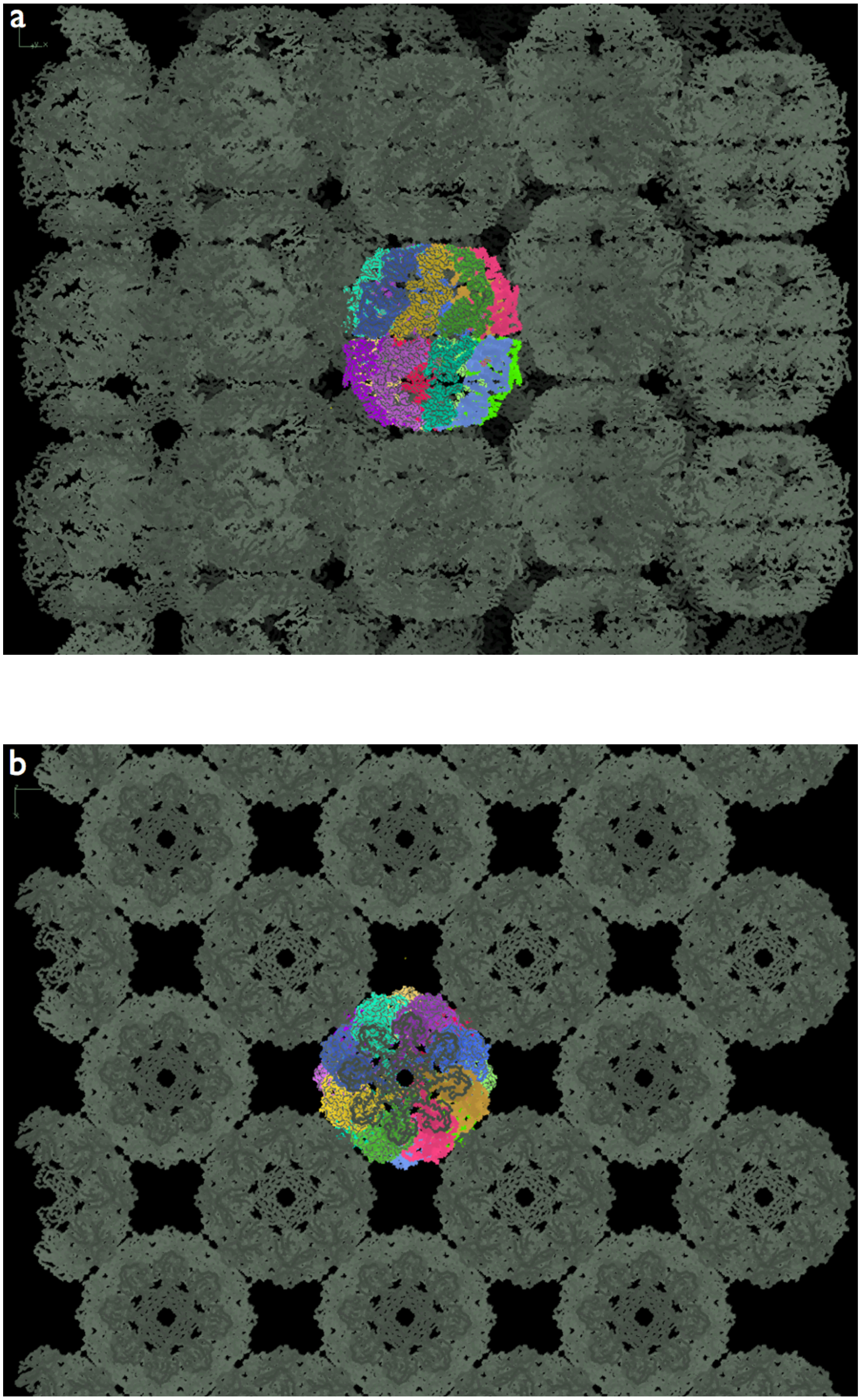

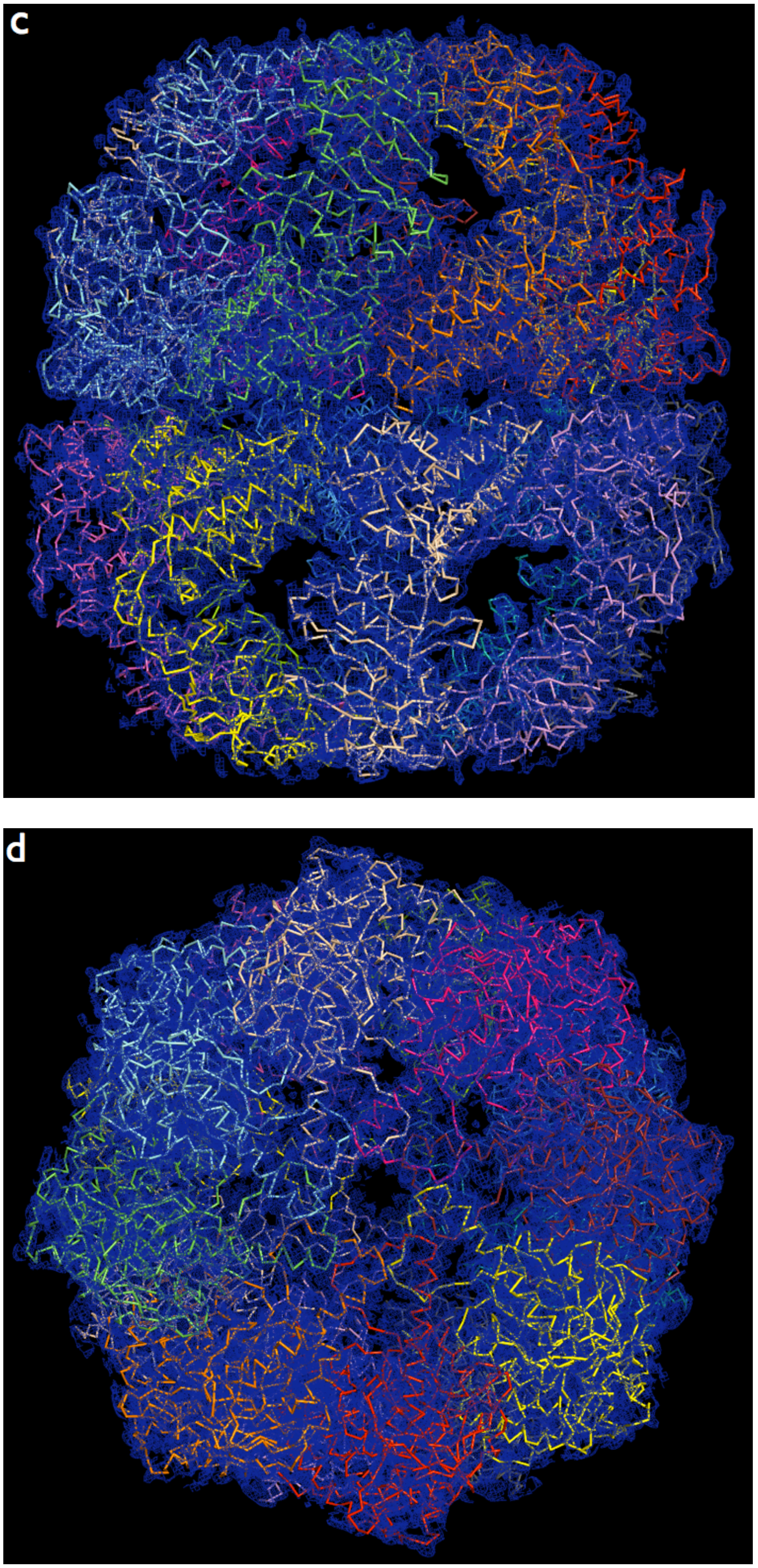
c
d

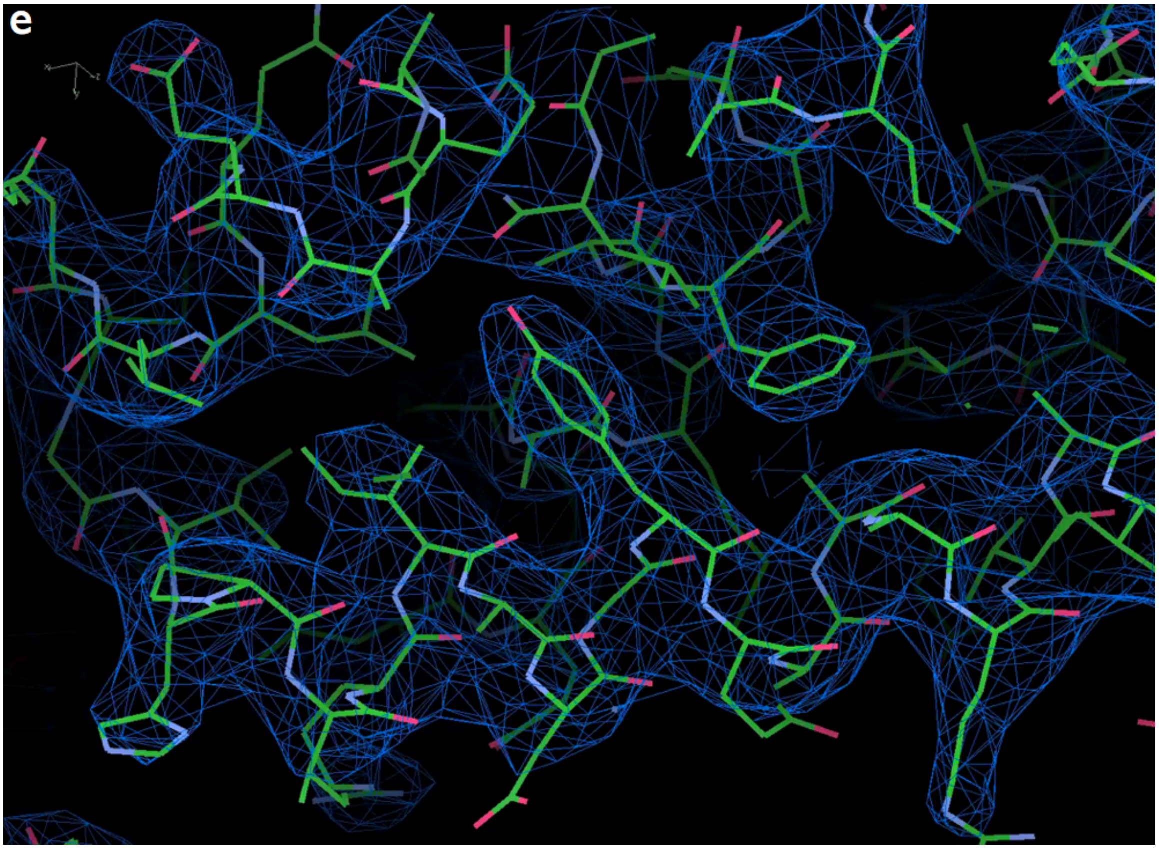

Supplementary Fig 2 – Crystal structure of the single CCT5 subunit shows the two back-to-back rings of eight subunits each, similar to the hetero-octameric human TRiC arrangement. **a,b)** Crystal packing showing the Side and Top view of CCT5 homo-octameric arrangements. **c,d,e)** Crystal structure of CCT5 solved at 3.5 Å resolution showing the experimental 2mFo-DFc electron density map contoured at 1.0 σ (blue) from the side and top view of the CCT5 double octameric rings arrangements and a zoom in at α-helices regions of equatorial domain, respectively.

Supplementary Fig 3

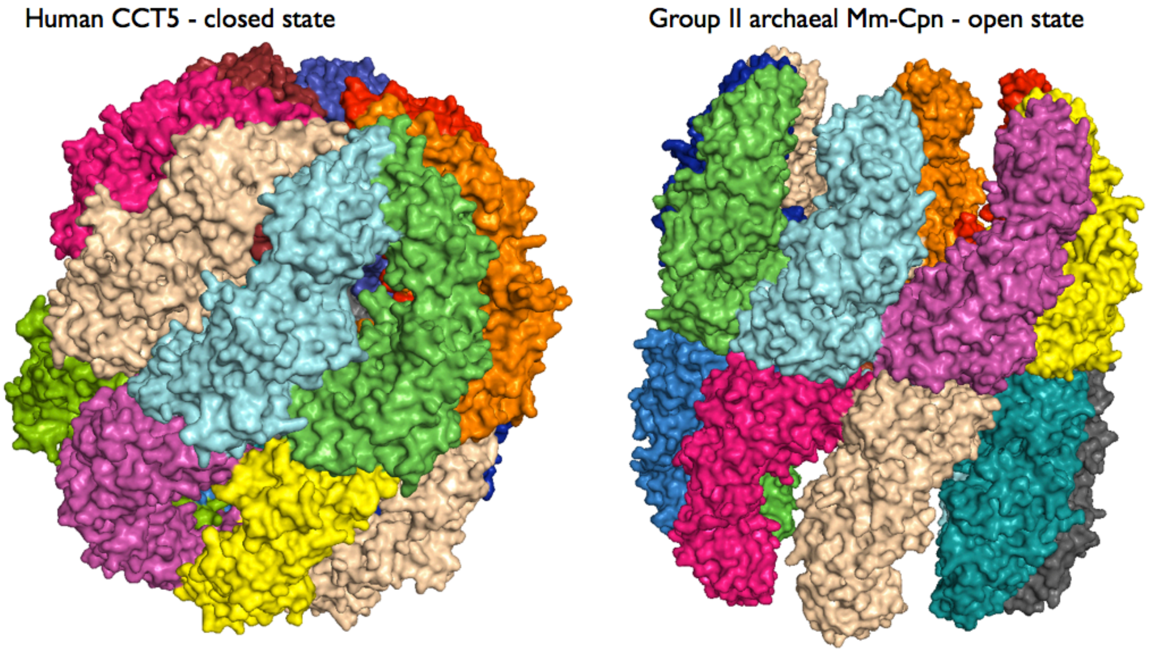

Supplementary Fig 3 – Homo-octameric double ring back-to-back arrangement of the CCT5 in a closed state and the group II chaperonin from *Methanococcus maripaludis* (Mm-Cpn) in an open state (PDB ID 3KFK) (Pereira *et al.*, 2010). The open state is associated with the peptide acceptor conformation that major conformation change upon the ATP hydrolysis and the chamber is closed to fold the protein substrates.

Supplementary Fig 4

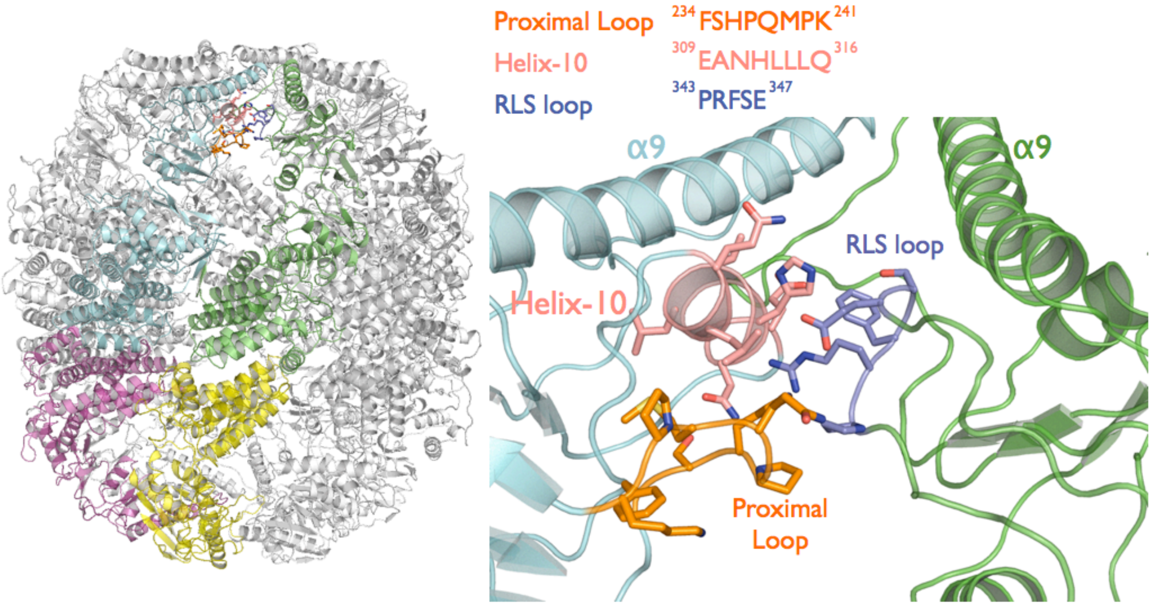

Supplementary Fig 4 – CCT5 ring arrangement showing substrate-binding site and release loop of substrate loop (rls-loop) contacting each other on the closed state observed for CCT5 in complex with ADP. Ring picture and a zoom in showing proximal loop (orange) and helix-10 (pink) of CCT5 subunit contacting the rls-loop (blue) from adjacent subunit at apical domain region of the ring.

References

Robert, X. & Gouet, P. Deciphering key features in protein structures with the new ENDscript server. *Nucl. Acids Res.* **42**, 320-324 (2014).

Pereira J.H. *et al.* Crystal structures of a group II chaperonin reveal the open and closed states associated with the protein folding cycle. *J. Biol. Chem.* **285**, 27958-27966 (2010).
